# Understanding Medication Adherence and Glycaemic Level in People With Type 2 Diabetes Across Countries: A Cross‐Sectional and Longitudinal Analysis of Medication Beliefs, Illness Perceptions, Resistance to Illness and Mood

**DOI:** 10.1002/edm2.70190

**Published:** 2026-03-03

**Authors:** Vivien Teo, John Weinman, Kai Zhen Yap, Shaun Eric Lopez, Anna Hodgkinson, Mark Chamley

**Affiliations:** ^1^ Institute of Pharmaceutical Sciences King's College London London UK; ^2^ Department of Pharmacy and Pharmaceutical Sciences National University of Singapore Singapore Singapore; ^3^ Division of Pharmacy Tan Tock Seng Hospital Singapore Singapore; ^4^ Lambeth Diabetes Intermediate Care Team, Guy's and St Thomas' NHS Foundation Trust London UK

**Keywords:** glycated haemoglobin, medication adherence, type 2 diabetes mellitus

## Abstract

**Background and Aims:**

Low medication adherence is prevalent in diabetes, and many adherence factors found are non‐modifiable by interventions. Adherence interventions have limited effectiveness due to a lack of tailoring and understanding of common modifiable factors. Hence, this study aimed to identify common and distinctive factors associated with medication adherence and glycaemic levels among people with type 2 diabetes in Singapore and the United Kingdom (UK) using cross‐sectional and longitudinal analyses of standardised measures.

**Methods:**

Medication adherence, medication beliefs, illness perceptions, mood, and glycaemic level were assessed using validated questionnaires and glycated haemoglobin (HbA1c) among 550 participants. Regressions and multilevel models investigated factors associated with baseline and 3–6‐month adherence and HbA1c.

**Results:**

Overall, participants with greater resistance to illness (*β* = −0.084, *p* = 0.031), medication concern (*β* = −0.069, *p* = 0.026) and reduced understanding of diabetes (*β* = 0.105, *p* = 0.053) had lower baseline adherence. Participants with greater adherence (*β* = −0.088, *p* = 0.006), personal control (*β* = −0.154, *p* < 0.001) and reduced emotional impact (*β* = 0.076, *p* = 0.008) had lower baseline HbA1c. Perceived treatment control was associated with follow‐up adherence (*β* = 0.140, *p* = 0.051), while personal control was associated with follow‐up HbA1c (*β* = −0.092, *p* = 0.009). In Singapore, resistance to illness (*β* = −0.110, *p* = 0.011) and medication concern (*β* = −0.101, *p* = 0.006) were significant adherence factors. Participants with greater testing treatment tendency (*β* = 0.146, *p* = 0.028) and lower personal control (*β* = −0.132, *p* = 0.006) had higher HbA1c. In the UK, sensitivity to medication was related to adherence (*β* = −0.262, *p* = 0.019), while adherence (*β* = −1.020, *p* = 0.040) and personal control (*β* = −1.803, *p* = 0.001) were linked to HbA1c.

**Conclusions:**

Common factors, such as resistance to illness and perceived personal control, may affect medication adherence or glycaemic level. Distinctive factors associated with medication adherence or glycaemic level include perceived sensitivity to medication and testing treatment. The common and distinctive factors identified could be used to support broadly applicable and locally relevant personalised intervention development.

## Graphical abstract

Common factors in the combined sample, such as resistance to illness and perceived personal control, may affect medication adherence or glycaemic level. Distinctive factors include perceived sensitivity to medication. The common and distinctive factors identified could be used to support broadly applicable and locally relevant personalised intervention development.

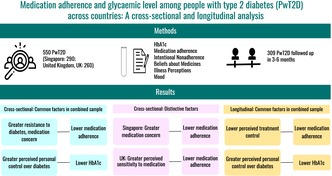

## 1 Introduction

Medication adherence is defined as the extent to which individuals' medication‐taking behaviour corresponds with agreed recommendations from healthcare professionals [1]. Low medication adherence observed among people with type 2 diabetes (PwT2D) is concerning, with meta‐analyses suggesting prevalence of approximately 30%–60% for oral and injectable medications [2, 3]. Current medication adherence interventions have limited effectiveness [4, 5], attributable to a lack of both investigation into individuals' specific adherence barriers and tailoring of intervention to the identified barrier [5]. For example, offering medication reminders to all PwT2D, including those who intentionally choose not to take their medications due to non‐acceptance of their diabetes, will understandably be ineffective due to a misidentification of the individual barrier and mismatch of the intervention.

Moreover, the potential effectiveness, relevance and transferability of interventions may be limited by current gaps in understanding the common adherence factors shared by PwT2D across different contexts. While systematic reviews may provide some insights on these common factors, the heterogeneous methods used to evaluate medication adherence and factors in these primary studies introduced variability in the measurement constructs and definitions [6, 7]. This inconsistency poses challenges for systematic reviews to differentiate common adherence factors from context‐specific factors in their aggregated findings. The lack of cross‐country comparison using standardised measures contributes to the challenges in understanding the common and distinctive factors.

Additionally, many adherence factors found so far are non‐modifiable, for example age [8]. Non‐modifiable factors may help to determine the groups at risk of having difficulties adhering to their medications. However, the effects of these non‐modifiable factors may be variable, as suggested by a systematic review of systematic reviews examining medication adherence studies [9]. Modifiable factors, on the other hand, are actionable through interventions. Among the modifiable factors, medication beliefs and illness perceptions have been shown to predict medication adherence consistently [10, 11]. A meta‐analysis of the Necessity‐Concerns Framework across countries demonstrated that people with lower adherence perceive medication as less necessary and have more concerns [12]. Adherence may also be affected by different dimensions of illness perception, as delineated in the Common‐Sense Model of illness representations (CSM) [13]. Moreover, qualitative studies found that people may reject their medications as they resist their illness and the notion of being ill [14, 15] due to the perception of medication‐taking as a reminder of poor health [15]. Research has also proposed the predictive role depressive symptoms play in low adherence to anti‐diabetic medications [16]. Understanding these modifiable factors among PwT2D across different contexts would facilitate the adaptation and tailoring of interventions to address them effectively.

Furthermore, the scarcity of longitudinal data and the predominance of cross‐sectional medication adherence studies affect our ability to capture the temporal relationships between the adherence factors and medication adherence [8, 9]. The lack of insights into these relationships limits prediction of future low adherence and development of interventions that are responsive to PwT2D's evolving adherence barriers.

Therefore, we aimed to explore the common and distinctive factors associated with medication adherence and glycaemic level among PwT2D across two countries using cross‐sectional and longitudinal analyses of standardised measures. We focused on modifiable factors, including medication beliefs, illness perceptions, resistance to illness, and mood, which are amenable to future personalised interventions. This study was conducted in Singapore and the United Kingdom (UK). Both countries are developed, multi‐ethnic societies representing several major ethnic groups from Asian and Western regions. Singapore is 74.0% Chinese, 13.5% Malays, and 9.0% Indians [17], while the UK is 81.7% White, with distinctions such as White British and Other Europeans, and 18.3% belong to black, Asian, mixed or other ethnic groups [18]. The age‐adjusted comparative prevalence of diabetes is projected to grow from 6.3% in 2021 to 7.5% in 2045 for the UK [19] and from 11.6% in 2021 to 14.3% in 2045 for Singapore [20]. The increasing prevalence of diabetes and varied ethnic compositions in both countries would potentially offer insights on the common factors relevant across different regions and populations. Our findings on the common factors, as well as the country‐specific distinctive factors, would support the development of broadly applicable and locally relevant personalised interventions.

## 2 Methodology

This study draws from our previous studies [21, 22] as part of our broader international study. The Singapore and UK studies were conducted in December 2021 to February 2023 and February to November 2024, respectively.

### 2.1 Participants

Participants were recruited during their regular clinic appointment.

Both studies utilised a similar methodology. PwT2D recruited were 21 years old or older, on anti‐diabetic medications, followed up with the endocrine department of a tertiary hospital in Singapore or diabetes intermediate care team in the UK, understood the study and language of the questionnaires (English or Mandarin in Singapore, English in the UK) and provided informed consent. Study follow‐up occurred after 3–6 months for those who consented, continued care with their provider, and had no hospitalisation since baseline. All participants were recruited in person and if they were eligible for the study follow‐up, they were followed up either in person or via telephone.

In Singapore, 290 participants were recruited, with 185 being followed up in 3–6 months. In the UK, 260 participants were recruited, of whom 124 completed the 3–6‐month follow‐up. Ethics approval was obtained from Singapore National Healthcare Group Domain Specific Review Board (2021/00397), National University of Singapore (2022–829), and London‐Chelsea National Health Service Research Ethics Committee (321743).

### 2.2 Materials and Measures

At baseline, all participants completed the following self‐reported questionnaires:

(a) *About you*: This 5‐item questionnaire captures sociodemographic information.

(b) *Visual Analogue Scale (VAS)*: This scale measures adherence levels between 0% and 100% in the last 3 months [23, 24].

(c) *Intentional Nonadherence Scale (INAS)*: This 22‐item scale includes four subscales on a 5‐point Likert scale, using the last 3 months as the reference period. They are “*Resisting illness and medication*”, “*Sensitivity to medication*”, “*Testing treatment*” and “*Inconvenience*” in the Singapore version [21], “*Resisting illness*”, “*Resisting medication*”, “*Testing treatment*” and “*Sensitivity to medication*” in the UK version [22].

(d) *Medication adherence report scale‐5 (MARS‐5)*: This 5‐item scale measures nonadherence on a 5‐point Likert scale over the last 3 months. Higher scores reflect greater adherence [25].

(e) *Beliefs about Medicine Questionnaires (BMQ)‐specific*: This 10‐item questionnaire measures beliefs about medication necessity and concerns across two subscales on a 5‐point Likert scale. Higher scores signify stronger perceived necessity and concerns [26, 27].

(f) *Brief Illness Perception Questionnaire (BIPQ)*: This 9‐item questionnaire measures different dimensions of illness perception on a scale of 0–10 for each dimension [28, 29].

(g) *Patient Health Questionnaire‐2 (PHQ‐2)*: This 2‐item scale assesses mood on a 4‐point Likert scale over the past 2 weeks. Higher scores indicate a greater likelihood of depression [30, 31].

Participants followed up in 3–6 months with repeated INAS and MARS‐5.

The questionnaires were translated and adapted for use in Singapore [21], while the study documents were reviewed by a Patient and Public Involvement group in the UK [22].

Clinical data were extracted from participants' medical records, including baseline and follow‐up glycated haemoglobin (HbA1c), years of diabetes, and anti‐diabetic medications.

The response rates in Singapore and the UK were 50%–60% [21] and 82% [22], respectively.

### 2.3 Statistical Analysis

Data were analysed with STATA 18. Descriptive statistics were presented as mean ± standard deviation (SD) or frequencies and percentages for categorical variables.

Measurement invariance of the INAS was assessed with Structural Equation Modelling (SEM) through Multi‐Group Confirmatory factor analysis (MGCFA) [32] to ascertain its psychometric equivalence across countries, as earlier studies identified different factor structures among PwT2D [21, 22]. Similar to other studies [33, 34], a reference factor structure was selected using CFA, followed by a four‐step invariance evaluation: configural equivalence of factor structure, metric equivalence of factor loadings, scalar equivalence of item intercepts, and strict equivalence of item residuals [32]. Invariance between countries is established when the likelihood‐ratio (LR) test (also known as the chi‐square difference test) yields a *p*‐value > 0.05, changes in the Comparative Fit Indexes (ΔCFI) ≤ 0.01, Tucker‐Lewis Index (ΔTLI) ≤ 0.01, Root Mean Square Error of Approximation (ΔRMSEA) ≤ 0.015, Standardised Root Mean Square Residual (ΔSRMR) ≤ 0.03 for metric invariance and ≤ 0.01 for scalar and strict invariance [35, 36]. Scalar invariance is necessary to analyse the INAS mean scores across countries [32].

Chi‐square test and independent *t*‐test examined the sociodemographic and clinical variables in both countries. Linear and quantile regression were performed for the parametric and non‐parametric self‐reported measures, respectively and were adjusted for the sociodemographic and clinical variables that showed statistically significant difference between countries. The median of MARS‐5 was used to categorise participants as having high and low adherence.

Common and distinctive factors are factors showing statistically significant associations with the outcomes in the analyses performed on the combined and individual samples, respectively. For the cross‐sectional analysis, multivariate quantile and linear regressions were conducted to identify factors associated with MARS‐5 and HbA1c, respectively. Self‐reported measures, sociodemographic and clinical variables that showed statistical significance in their respective univariate regressions were entered into the multivariate regressions, while also adjusting for medication type, which was considered clinically relevant. Multilevel models were used to identify factors associated with the follow‐up MARS‐5 and HbA1c in 3–6 months in the combined sample adjusted for country, baseline covariates, interval between baseline and follow‐up, as well as the consistency in the questionnaire administration mode. Sensitivity analysis was performed to check for baseline differences in the combined sample of participants who were and were not followed up in 3–6 months.

Mediation analyses using SEM with maximum likelihood estimation were employed to explore potential relationships between factors associated with medication nonadherence, using baseline and follow‐up MARS‐5 as the outcome. The hypotheses for the mediation models were based on three considerations: (1) Variables that showed statistically significant associations with MARS‐5 and medication belief in the multivariate regressions; (2) an earlier study that proposed medication beliefs as mediators in medication adherence among PwT2D [10]; and (3) the Extended CSM, which proposes that medication beliefs, illness perceptions and medication adherence are interconnected [37]. In the mediation analysis with follow‐up MARS‐5 as the primary outcome, autoregressive paths were added between baseline and follow‐up MARS‐5 to account for stability and changes over time [38]. The consistency of questionnaire administration mode at follow‐up and the interval between baseline and follow‐up were also adjusted for. Model fit was considered acceptable when CFI > 0.9, TLI > 0.9, RMSEA < 0.08 and SRMR < 0.08 [39, 40]. Standardised beta coefficients (*β*) were obtained to facilitate interpretation across different variables. Monte Carlo simulation with 1000 replications was performed retrospectively to ensure that the mediation pathways have at least 80% power [41].

## 3 Results

### 3.1 Measurement Invariance of the INAS

The UK factor structure was used as the reference to evaluate measurement invariance of the INAS, as it exhibited a better model fit than the Singapore factor structure (UK factor structure versus Singapore factor structure: RMSEA = 0.105 vs. 0.118, CFI = 0.923 vs. 0.898, TLI = 0.909 vs. 0.880, SRMR = 0.043 vs. 0.060). The results can be found in Appendix S1. The items for the Singapore and UK factor structure, along with the questionnaire guide for Singapore INAS, are in Appendix S2.

### 3.2 Sociodemographic, Clinical Variables and Self‐Reported Measures

The sociodemographic and clinical characteristics are shown in Table 1.

**TABLE 1:** Sociodemographic, clinical variables and self‐report measures at baseline.

Variable	Total (*n* = 550)	Singapore (*n* = 290)	UK (*n* = 260)	*p*
Age, mean ± SD	59.35 ± 11.82	57.94 ± 11.78	60.92 ± 11.67	0.0031
Sex, *n* (%)				0.001
Male	310 (56.36)	182 (62.76)	128 (49.23)	
Female	240 (43.64)	108 (37.24)	132 (50.77)	
Ethnicity, *n* (%)				< 0.001
White	74 (13.45)	NA	74 (28.46)	
Black/Black British	118 (21.45)	NA	118 (45.38)	
Asian/Asian British	23 (4.18)	NA	23 (8.85)	
Mixed	14 (2.55)	NA	14 (5.38)	
Others	33 (6.00)	5 (1.72)	28 (10.77)	
Chinese—Singapore	193 (35.09)	193 (66.55)	NA	
Malay—Singapore	42 (7.64)	42 (14.48)	NA	
Indian—Singapore	50 (9.09)	50 (17.24)	NA	
Declined to answer	3 (0.55)	0	3 (1.15)	
Highest education level, *n* (%)				< 0.001
No formal education	5 (0.91)	1 (0.34)	4 (1.54)	
Primary school/lower	74 (13.45)	56 (19.31)	18 (6.92)	
Secondary school	228 (41.45)	135 (46.55)	93 (35.77)	
A‐level/diploma	126 (22.91)	67 (23.10)	59 (22.69)	
Degree/higher	116 (21.09)	31 (10.69)	85 (32.69)	
Declined to answer	1 (0.18)	0	1 (0.38)	
Relationship status, *n* (%)				< 0.001
Single	151 (27.45)	61 (21.03)	90 (34.62)	
Married	308 (56.00)	196 (67.59)	112 (43.08)	
Separated/divorced/widowed	79 (14.36)	31 (10.69)	48 (18.46)	
Others	11 (2.00)	2 (0.69)	9 (3.46)	
Declined to answer	1 (0.18)	0 (0.00)	1 (0.38)	
Manage own medication, *n* (%)				0.08
No	21 (3.82)	15 (5.17)	6 (2.31)	
Yes	529 (96.18)	275 (94.83)	254 (97.69)	
Number of chronic diseases^a^, mean ± SD	2.99 ± 1.42	3.52 ± 1.40	2.39 ± 1.20	< 0.001
Common types of chronic diseases^a^, *n* (%)				
Hypertension	347 (63.09)	214 (73.79)	133 (51.15)	< 0.001
Hyperlipidaemia	260 (47.27)	244 (84.14)	6 (6.15)	< 0.001
Chronic kidney disease	111 (20.18)	54 (18.62)	57 (21.92)	0.335
Ischaemic heart disease	103 (18.73)	71 (24.48)	32 (12.31)	< 0.001
Osteoarthritis	62 (11.27)	37 (12.76)	25 (9.62)	0.245
Medication type, *n* (%)				0.425
Oral only	162 (29.45)	89 (30.69)	73 (28.08)	
Injectables only	18 (3.27)	7 (2.41)	11 (4.23)	
Oral + Injectables	370 (67.27)	194 (66.90)	176 (67.69)	
Years of diabetes, *n* (%)				< 0.001
Unspecified record	81 (14.73)	70 (24.14)	11 (4.23)	
≤ 1 year	27 (4.91)	15 (5.17)	12 (4.62)	
> 1 year, ≤ 5 years	48 (8.73)	25 (8.62)	23 (8.85)	
> 5 years, < 10 years	69 (12.55)	35 (12.07)	34 (13.08)	
10–19 years	188 (34.18)	80 (27.59)	108 (41.54)	
≥ 20 years	137 (24.91)	65 (22.41)	72 (27.69)	
HbA1c (%), mean ± SD	8.8 ± 1.9	8.3 ± 1.7	9.3 ± 1.9	< 0.001
HbA1c (mmol/mol), mean ± SD	72 ± 20	67 ± 19	78 ± 20	< 0.001
Self‐report measures (possible range), adjusted mean/median ± SE				
MARS‐5 (5–25)^b^	23.67 ± 0.11	23.80 ± 0.59	23.54 ± 0.66	0.831
VAS (0–100)^b^	95.51 ± 0.78	96.55 ± 4.36	94.35 ± 4.85	0.809
INAS‐Resisting illness (1–35)^c^	13.89 ± 0.20	17.20 ± 1.12	10.19 ± 1.25	0.003
INAS‐Resisting medication (1–25)^c^	9.69 ± 0.14	11.34 ± 0.79	7.84 ± 0.88	0.034
INAS‐Testing treatment (1–15)^c^	5.70 ± 0.09	5.96 ± 0.49	5.41 ± 0.55	0.59
INAS‐Sensitivity to medication (1–15) ^+^	5.91 ± 0.09	7.01 ± 0.53	4.68 ± 0.58	0.033
BMQ‐Necessity (5–25)^c^	16.72 ± 0.15	16.71 ± 0.86	16.73 ± 0.96	0.989
BMQ‐Concern (5–25)^c^	13.61 ± 0.15	14.22 ± 0.86	12.94 ± 0.96	0.475
BIPQ1‐Consequence (0–10)^c^	5.64 ± 0.11	5.30 ± 0.62	6.00 ± 0.69	0.588
BIPQ2‐Timeline (0–10)^b^	8.20 ± 0.14	8.08 ± 0.76	8.33 ± 0.84	0.874
BIPQ3‐Personal control (0–10)^c^	6.83 ± 0.09	6.26 ± 0.51	7.46 ± 0.56	0.256
BIPQ4‐Treatment control (0–10)^b^	8.05 ± 0.11	6.75 ± 0.61	9.51 ± 0.67	0.029
BIPQ5‐Identity (0–10)^c^	5.40 ± 0.11	4.65 ± 0.62	6.22 ± 0.69	0.222
BIPQ6‐Concern (0–10)^b^	8.07 ± 0.17	7.00 ± 0.94	9.26 ± 1.05	0.249
BIPQ7‐Understanding (0–10)^b^	8.11 ± 0.11	8.24 ± 0.63	7.97 ± 0.70	0.836
BIPQ8‐Emotional response (0–10)^c^	5.30 ± 0.13	5.99 ± 0.71	4.54 ± 0.79	0.329
PHQ‐2 (0–6)^b^	0.21 ± 0.07	0.21 ± 0.36	0.21 ± 0.40	1.00

Abbreviations: BIPQ, Brief Illness Perception Questionnaire; BMQ, Beliefs about Medicine Questionnaires; INAS, Intentional Nonadherence Scale; MARS‐5, medication adherence report scale‐5; NA, non‐applicable; PHQ‐2, Patient Health Questionnaire‐2; VAS, Visual Analogue Scale.

^a^ Limited information on chronic diseases.

^b^ Adjusted median ± standard error (SE).

^c^ Adjusted mean ± SE.

Statistically significant differences were observed in age, sex, ethnicity, highest education level, relationship status, years of diabetes, number of chronic diseases and baseline HbA1c between countries. Overall, the mean ± SD age of the participants was 59.4 ± 11.8 years and 56.4% of the participants were male. Most participants had at least secondary school education (85.6%). The mean ± SD HbA1c of the participants was 8.8% ± 1.9% (72 ± 20 mmol/mol). The HbA1c of the participants in Singapore was lower than that in the UK sample (8.3% ± 1.7% vs. 9.3% ± 1.9%; 67 ± 19 mmol/mol vs. 78 ± 20 mmol/mol).

The self‐reported measures presented in Table 1 were adjusted for age, sex, ethnicity, highest education level, relationship status, years of diabetes and HbA1c, all of which showed statistically significant differences between countries. In the combined sample, the adjusted median ± standard error (SE) of MARS‐5 score was 23.7 ± 0.1, with no significant difference between countries (*p* = 0.831). The MARS‐5 median of 24 was used to categorise participants with high (MARS‐5 = 25) and low medication adherence (MARS‐5 ≤ 24). Overall, 60.7% of the participants had low adherence, while its proportion in Singapore and the UK was 63.1% and 58.1%, respectively.

Most self‐reported measures did not show statistically significant differences across countries. The measures that showed significant differences between countries were INAS‐Resisting illness (Singapore: 17.2 ± 1.1, UK: 10.2 ± 1.3, *p* = 0.003), INAS‐Resisting medication (Singapore: 11.3 ± 0.8, UK: 7.8 ± 0.9, *p* = 0.034), INAS‐Sensitivity to medication (Singapore: 7.0 ± 0.5, UK: 4.7 ± 0.6, *p* = 0.033) and BIPQ4‐Treatment Control (Singapore: 6.8 ± 0.6, UK: 9.5 ± 0.7, *p* = 0.029).

### 3.3 Factors Associated With Baseline MARS‐5 and HbA1c

Table 2 presents the multivariate regressions of factors associated with baseline MARS‐5 and HbA1c in the combined sample. INAS‐Resisting illness (*β* = −0.084, *p* = 0.031), BMQ‐Concern (*β* = −0.069, *p* = 0.026) and BIPQ7‐Understanding (*β* = 0.105, *p* = 0.053) were significantly associated with baseline MARS‐5, after adjusting for country, age, ethnicity, years of diabetes and medication type. On the other hand, MARS‐5 (*β* = −0.09, *p* = 0.006), BIPQ3‐Personal control (*β* = −0.154, *p* < 0.001) and BIPQ8‐Emotional response (*β* = 0.076, *p* = 0.008) showed significant associations with baseline HbA1c, after controlling for country, ethnicity, highest education level, years of diabetes and medication type.

**TABLE 2:** Multivariate regression on factors associated with baseline MARS‐5 and HbA1c in the combined sample (*n* = 550).

Outcome	Baseline MARS‐5		Baseline HbA1c	
Variables	Coefficient	*p*	Coefficient	*p*
Country	Reference: Singapore		Reference: Singapore	
UK	−0.444	0.697	0.218	0.795
Age	0.051	< 0.001	NA	NA
Ethnicity	Reference: White		Reference: White	
Black/Black British	0.247	0.484	0.113	0.667
Asian/Asian British	1.129	0.045	−0.357	0.396
Mixed	−0.275	0.681	−0.109	0.825
Others	0.603	0.247	0.121	0.754
Chinese—Singapore	0.148	0.900	−0.868	0.319
Malay—Singapore	0.269	0.825	−0.638	0.479
Indian—Singapore	−0.098	0.936	−0.980	0.273
Declined to answer	−1.597	0.243	1.423	0.244
Highest education level	NA	NA	Reference: No formal education	
Primary school/lower	NA	NA	−1.826	0.023
Secondary school	NA	NA	−2.039	0.010
A‐level/diploma	NA	NA	−2.107	0.008
Degree/higher	NA	NA	−2.244	0.005
Declined to answer	NA	NA	−6.706	0.003
Years of diabetes (medical records)	Reference: ≥ 20 years		Reference: ≥ 20 years	
Unspecified record	0.124	0.720	−0.100	0.690
≤ 1 year	0.907	0.077	−0.618	0.094
> 1 year, ≤ 5 years	0.178	0.666	−0.415	0.152
> 5 years, < 10 years	0.924	0.011	0.047	0.856
10–19 years	0.564	0.037	0.062	0.748
Medication type	Reference: Oral only		Reference: Oral only	
Injectables only	0.471	0.420	0.787	0.067
Oral + Injectable	−0.360	0.108	0.706	< 0.001
MARS‐5	NA	NA	−0.088	0.006
INAS‐Resisting illness	−0.084	0.031	0.038	0.121
INAS‐Resisting medication	0.013	0.837	NA	NA
INAS‐Testing treatment	−0.088	0.229	0.006	0.918
INAS‐Sensitivity to medication	−0.087	0.221	0.008	0.867
BMQ‐Concern	−0.069	0.026	−0.009	0.706
BIPQ1‐Consequence	NA	NA	−0.046	0.187
BIPQ3‐Personal control	0.045	0.386	−0.154	< 0.001
BIPQ4‐Treatment control	0.075	0.215	NA	NA
BIPQ5‐Identity	NA	NA	−0.002	0.949
BIPQ6‐Concern	NA	NA	0.051	0.127
BIPQ7‐Understanding	0.105	0.053	NA	NA
BIPQ8‐Emotional response	−0.019	0.591	0.076	0.008

Abbreviations: BIPQ, Brief Illness Perception Questionnaire; BMQ, Beliefs about Medicine Questionnaires; INAS, Intentional Nonadherence Scale; MARS‐5, Medication adherence report scale‐5; NA, non‐applicable.

Appendix S3 shows the multivariate regressions of factors that showed an association with baseline MARS‐5 and HbA1c in the Singapore sample. INAS‐Resisting illness (*β* = −0.110, *p* = 0.011), and BMQ‐Concern (*β* = −0.101, *p* = 0.006) were significantly and negatively associated with MARS‐5, after adjusting for age, years of diabetes and medication type. INAS‐Testing treatment (*β* = 0.146, *p* = 0.028) and BIPQ3‐Personal control (*β* = −0.132, *p* = 0.006) demonstrated significant associations with HbA1c in Singapore, after adjusting for ethnicity and medication type.

Appendix S4 shows the multivariate regressions of factors related to baseline MARS‐5 and HbA1c in the UK sample. INAS‐Sensitivity to medication (*β* = −0.262, *p* = 0.019) was the only self‐reported measure associated with MARS‐5 significantly, after controlling for age, ethnicity, relationship status, medication type and highest education level. MARS‐5 (*β* = −1.020, *p* = 0.040) and BIPQ3‐Personal control (*β* = −1.803, *p* = 0.001) were significantly and negatively associated with HbA1c in the UK after adjusting for the highest education level and medication type.

Appendices S5 and S6 present the univariate regressions of factors related to baseline MARS‐5 and Hba1c, respectively for all the samples.

### 3.4 Factors Associated With Follow‐Up MARS‐5 and HbA1c

Table 3 summarises the multilevel models of factors that showed an association with follow‐up MARS‐5 and HbA1c in the combined sample. This longitudinal analysis was not conducted for each country, as country did not show statistically significant effects in the earlier baseline analysis in Table 2.

**TABLE 3:** Multilevel model on factors associated with follow‐up MARS‐5 and HbA1c in the combined sample (*n* = 309).

Outcome	Follow‐up MARS‐5		Follow‐up HbA1c	
Variables	Coefficient	*p*	Coefficient	*p*
Country	Reference: Singapore		Reference: Singapore	
UK	1.260	0.398	0.880	0.368
Age	0.048	< 0.001	NA	NA
Ethnicity	Reference: White		Reference: White	
Black/Black British	−0.092	0.834	0.206	0.460
Asian/Asian British	1.096	0.001	0.250	0.545
Mixed	−2.211	0.096	1.029	0.090
Others	0.602	0.546	0.174	0.664
Chinese—Singapore	1.843	0.210	0.240	0.809
Malay—Singapore	2.473	0.107	0.209	0.838
Indian—Singapore	2.214	0.128	0.074	0.942
Highest education level	NA	NA	Reference: No formal education	
Primary school/lower	NA	NA	−0.851	0.365
Secondary school	NA	NA	−0.793	0.393
A‐level/diploma	NA	NA	−1.074	0.252
Degree/higher	NA	NA	−1.106	0.238
Years of diabetes (medical records)	Reference: ≥ 20 years		Reference: ≥ 20 years	
Unspecified record	0.278	0.359	0.162	0.525
≤ 1 year	−0.712	0.469	−0.519	0.150
> 1 year, ≤ 5 years	1.045	0.023	0.014	0.965
> 5 years, < 10 years	1.114	< 0.001	0.064	0.808
10–19 years	0.817	0.003	0.238	0.218
Medication type at follow‐up	Reference: Oral only		Reference: Oral only	
Injectables only	−0.607	0.359	0.608	0.173
Oral + Injectable	−0.377	0.124	0.872	< 0.001
Interval between baseline and follow‐up	0.002	0.559	−0.004	0.101
Questionnaire administration mode at follow‐up	Reference: Different from baseline		Reference: Different from baseline	
Same as baseline	−0.135	0.536	−0.053	0.739
MARS‐5	NA	NA	−0.056	0.128
INAS‐Resisting illness	−0.078	0.186	−0.009	0.736
INAS‐Resisting medication	0.219	0.072	NA	NA
INAS‐Testing treatment	−0.264	0.120	−0.002	0.972
INAS‐Sensitivity to medication	−0.074	0.557	0.064	0.244
BMQ‐Concern	−0.022	0.545	0.001	0.959
BIPQ1‐Consequence	NA	NA	−0.060	0.091
BIPQ3‐Personal control	−0.014	0.800	−0.092	0.009
BIPQ4‐Treatment control	0.140	0.051	NA	NA
BIPQ5‐Identity	NA	NA	0.046	0.192
BIPQ6‐Concern	NA	NA	0.041	0.249
BIPQ7‐Understanding	0.062	0.215	NA	NA
BIPQ8‐Emotional response	−0.040	0.372	0.029	0.311

Abbreviations: BIPQ, Brief Illness Perception Questionnaire; BMQ, Beliefs about Medicine Questionnaires; INAS, Intentional Nonadherence Scale; MARS‐5, Medication adherence report scale‐5; NA, non‐applicable.

The multilevel model for MARS‐5 was adjusted for country, age, ethnicity, years of diabetes, medication type at follow‐up, interval between baseline and follow‐up, as well as the consistency of questionnaire administration mode at follow‐up. After accounting for the clustering effect of repeated MARS‐5 within individual participants over time, BIPQ4‐Treatment control was the only self‐reported measure that showed a statistically significant association with follow‐up MARS‐5 (*β* = 0.140, *p* = 0.051).

The multilevel model for HbA1c was adjusted for country, ethnicity, education level, years of diabetes, medication type at follow‐up, interval between baseline and follow‐up, as well as the consistency of questionnaire administration mode at follow‐up. After adjusting for the repeated HbA1c within individual participants over time, BIPQ3‐Personal control was found to be significantly associated with follow‐up HbA1c (*β* = −0.092, *p* = 0.009). The sensitivity analysis on the combined sample in Appendix S7 found that baseline characteristics and self‐report results are largely similar in participants who were and were not followed up in 3–6 months, except for some differences detected in ethnicity and relationship status. Among those who were not followed up, 28.6% were Chinese from the Singapore sample and 27.4% were Black or Black British from the UK sample. In contrast, among those who were followed up, 40.1% were Chinese from the Singapore sample and 16.8% were Black or Black British from the UK sample. Participants who were not followed up included 49.4% married individuals, while participants who were followed up included 61.2% married individuals.

### 3.5 Mediation

Appendix S8 is the conceptual mediation model developed with MARS‐5 as the outcome, BMQ‐Concern hypothesised as a mediator based on an earlier study [10] and our multivariate regression results. The mediation analyses were conducted on the combined and Singapore sample, but not on the UK sample, as BMQ‐Concern was significantly associated with MARS‐5 in the former samples only. The potential predictors of BMQ‐Concern in the combined and Singapore samples are presented in Appendices S9 and S10, respectively.

Table 4 summarises mediation results with over 80% statistical power, adjusted for age, which consistently showed significant association with MARS‐5 across all samples. In the combined sample, BMQ‐Concern significantly mediated the effects of BIPQ1‐Consequence (*β* = −0.02, *p* < 0.001), BIPQ4‐Treatment control (*β* = 0.01, *p* < 0.001), BIPQ6‐Concerns (*β* = −0.01, *p* < 0.001), and BIPQ8‐Emotional response (*β* = −0.02, *p* < 0.001) on MARS‐5. In the Singapore sample, BMQ‐Concern significantly mediated the effects of INAS‐Resisting illness (*β* = −0.05, *p* = 0.01) and INAS‐Resisting medication (*β* = −0.08, *p* < 0.001). About 28% of the total effect of INAS‐Resisting illness on MARS‐5 was mediated through BMQ‐Concern.

**TABLE 4:** Mediation analysis with baseline MARS‐5 as the outcome and BMQ‐concern as the mediator.

Predictor	Sample	Power	RMSEA	CFI	TLI	SRMR	*β*	SE	95% CI		*p*
BIPQ1‐Consequence	Combined	81.68	0.06	0.93	0.91	0.05	−0.02	0.00	−0.03	−0.01	< 0.001
BIPQ4‐Treatment control	Combined	82.30	0.07	0.92	0.90	0.05	0.01	0.00	0.01	0.02	< 0.001
BIPQ6‐Concern	Combined	80.92	0.06	0.94	0.92	0.04	−0.01	0.00	−0.02	−0.01	< 0.001
BIPQ7‐Understanding	Combined	89.69	0.07	0.91	0.88	0.05	0.00	0.00	0.00	0.01	0.14
BIPQ8‐Emotional response	Combined	82.46	0.06	0.94	0.92	0.04	−0.02	0.00	−0.02	−0.01	< 0.001
INAS‐Resisting illness	Singapore	85.70	0.08	0.90	0.88	0.07	−0.05	0.02	−0.09	−0.01	0.01
INAS‐Resisting medication	Singapore	100.00	0.07	0.93	0.91	0.08	−0.08	0.02	−0.13	−0.03	< 0.001

Abbreviations: BIPQ, Brief Illness Perception Questionnaire; BMQ, Beliefs about Medicine Questionnaires; CFI, Comparative Fit Index; INAS, Intentional Nonadherence Scale; MARS‐5, medication adherence report scale‐5. RMSEA, Root Mean Square Error of Approximation; SE, standard error; SRMR, Standardised Root Mean Squared Residual; TLI, Tucker‐Lewis Index; *β*, standardised beta.

All the mediation pathways with follow‐up MARS‐5 as the outcome had less than 80% statistical power.

### 3.6 Summarised Results

Given the detailed nature of the study findings, Table 5 provides an overview of the factors significantly associated with baseline and/or follow‐up MARS‐5 and HbA1c in Singapore, the UK and combined samples.

**TABLE 5:** Overview of the statistically significant factors affecting MARS‐5 and HbA1c in different samples.

Outcome	Statistically significant factors		
	Combined	Singapore	UK
Baseline MARS‐5	– Age – Ethnicity – Years of diabetes – INAS‐Resisting illness – BMQ‐Concern – BIPQ7‐Understanding	– Age – INAS‐Resisting illness – BMQ‐Concern	– Age – Medication type – INAS‐Sensitivity to medication
Baseline HbA1c	– Highest education level – Medication type – MARS‐5 – BIPQ3‐Personal control – BIPQ8‐Emotional response	– Medication type – INAS‐Testing treatment – BIPQ3‐Personal control	– Highest education level – MARS‐5 – BIPQ3‐Personal control
Follow‐up MARS‐5	– Age – Ethnicity – Years of diabetes – BIPQ4‐Treatment control	N/A	N/A
Follow‐up HbA1c	– Medication type – BIPQ3‐Personal control	N/A	N/A
Mediation with baseline MARS‐5 (outcome) and BMQ‐Concern (mediator)	– BIPQ1‐Consequence – BIPQ4‐Treatment control – BIPQ6‐Concern – BIPQ8‐Emotional response	– INAS‐Resisting illness – INAS‐Resisting medication	N/A

Abbreviations: BIPQ, Brief Illness Perception Questionnaire; BMQ, Beliefs about Medicine Questionnaires; INAS, Intentional Nonadherence Scale; MARS‐5, Medication adherence report scale‐5; NA, non‐applicable.

## 4 Discussion

Generally, this study identified that people who report higher resistance to illness (INAS), higher perceived sensitivity to medication (INAS), greater medication concerns (BMQ), lower perceived helpfulness of treatment (BIPQ4), and reduced perceived understanding of diabetes (BIPQ7) were found to be less adherent to their medications in the combined and/or individual sample at baseline or follow‐up. On the other hand, people who report lower medication adherence (MARS‐5), greater tendency to test their treatment (INAS), lower perceived control over their diabetes (BIPQ3), and/or greater emotional impact of diabetes (BIPQ8) tend to have higher HbA1c in the combined and/or individual sample at baseline and/or follow‐up.

Potential mediation mechanisms were identified with BMQ‐concern as the mediator. PwT2D who have greater perceived serious consequences from diabetes (BIPQ1), lower perceived helpfulness of treatment (BIPQ4), greater concerns about diabetes (BIPQ6), or greater emotional impact of diabetes (BIPQ8) may have greater concerns about their medications (BMQ‐Concern) too, resulting in low medication adherence.

The overall adherence level (adjusted median = 23.7) in our study was comparable to that reported in a previous study involving PwT2D (median = 24), as was the overall proportion of PwT2D with low adherence (60.7% versus 57.1%) [42]. The slightly higher proportion of medication nonadherence observed in our study may be attributed to the inclusion of PwT2D on oral and/or injectable medications with a more complex treatment plan, as the previous study only investigated adherence to oral anti‐diabetic medications [42].

### 4.1 Common Factors

Resisting illness appeared to be a prominent factor associated with baseline medication adherence. This finding was consistent with an earlier synthesis highlighting that PwT2D who do not accept their diabetes are also not likely to accept their medication [14]. This barrier was observed in over 40% of the studies reviewed [14] and may be because medication is an unwelcome reminder of them having diabetes [43]. Acceptance and Commitment Therapy may be a strategy for addressing this common barrier. It is a newer form of cognitive behavioural therapy, which supports people to engage and live alongside their challenging circumstances while adopting health behaviours, such as medication adherence that are aligned with their life goals (psychological flexibility) [44].

Although participants had greater perceived necessity for medications (BMQ‐Necessity) than medication concern (BMQ‐Concern) across all samples, only BMQ‐Concern and not BMQ‐Necessity was significantly associated with medication adherence. This seemed to suggest that PwT2D's concerns about their medications may outweigh their beliefs about the necessity of their medications, resulting in nonadherence. This could be due to the nature of diabetes and its medications. As compared to the generally asymptomatic and chronic nature of diabetes, medication side effects may be more immediate and tangible to PwT2D. Concern about medication side effects was also identified as a major adherence barrier in a qualitative synthesis as it was reported in 19 out of the 22 studies included [14]. Many PwT2D expressed fear and concerns about having diarrhoea from metformin, hypoglycaemia, and feeling very sick after taking their medications [14]. Hence, addressing medication concerns may be more effective than reinforcing necessity beliefs for improving adherence among PwT2D.

In addition to being a common barrier, medication concern is also a significant mediator in pathways connecting to medication adherence. While an earlier study suggested specific necessity and general beliefs of medications as mediators [10], our analyses proposed medication concern as a mediator instead. This was based on our regression results and the Extended CSM, which proposes that people's illness perceptions and medication beliefs interact as they try to formulate a coherent understanding and coping strategy (medication adherence/nonadherence) in the face of their diabetes [37]. They may generalise their concern about their diabetes to their medications and have difficulty distinguishing diabetes‐related symptoms and complications from medication side effects [14]. Therefore, future interventions are recommended to target both illness perception and medication concern, addressing both the distal and proximal predictors of medication adherence.

The perceived personal control over diabetes (BIPQ3) consistently emerges as a significant common factor associated with baseline and follow‐up HbA1c across all samples. Although the regression coefficient of −0.154 for BIPQ3‐Personal control in the combined sample may appear less clinically significant than the 0.5%–1.0% reduction in HbA1c aimed by some researchers and clinicians, HbA1c target levels are individualised [45, 46]. Modest associations could still be clinically meaningful, for example in a multi‐pronged strategy addressing multiple factors concurrently. The relationship between personal control and HbA1c may be understood through the CSM, which posits that individuals' cognitive representations of their illness shape their self‐regulatory behaviours [47]. PwT2D who perceive themselves as having control over their diabetes are more likely to engage in activities that can improve their HbA1c, such as increasing physical activity and adopting a healthier diet. When PwT2D see that these self‐regulatory mechanisms work as expected, such as through improved HbA1c, this reinforces their beliefs in the effectiveness of these actions [47]. The importance of personal control was also underscored in another study, which proposed that personal control is associated with HbA1c and significantly mediates the effects of diabetes‐related distress on HbA1c among PwT2D [48]. Considering the pronounced effects demonstrated in both prior research and our study, future research should focus on developing interventions aimed at empowering PwT2D to enhance their sense of personal control over diabetes.

Notably, the sense of personal control over diabetes (BIPQ3) among PwT2D has a stronger effect on the baseline HbA1c in the UK (*β* = −1.803) than in Singapore (*β* = −0.132). This difference may stem from the UK's more individualistic culture, which has a greater emphasis on self‐agency, making people more inclined to perceive diabetes as a challenge to overcome [49]. This is in contrast with Singapore's more collectivistic culture, in which people may be more dependent on family or social initiatives and more likely to perceive diabetes with pessimism [49]. While perceived personal control is a common factor associated with HbA1c across countries, interventions targeting this factor may have varying levels of success due to its different magnitude of association in each context.

The relationship identified between BIPQ3‐Personal control and follow‐up HbA1c, as well as BIPQ4‐Treatment control and follow‐up medication adherence could be understood based on the construct of each BIPQ item. BIPQ3‐Personal control was reasonably associated with HbA1c as it was related to PwT2D's overall perceived control over their diabetes. This included other aspects of their diabetes management, such as diet and physical activity that could also influence their HbA1c. On the other hand, BIPQ4‐Treatment control was more specific to PwT2D's perceived helpfulness of their treatments, which was more centred on their medications, and therefore was associated with medication adherence. The absence of a significant association between BIPQ4‐Treatment control and medication adherence at baseline may have resulted from the temporal gap in ascertaining whether the medication was helpful. Therefore, longitudinal analysis was helpful in revealing potential temporal effects of predictors on medication adherence and HbA1c, which cannot be captured in a cross‐sectional study.

### 4.2 Distinctive Factors

In Singapore, medication concerns may be more related to the broader negative beliefs about medications (BMQ‐concern), such as scepticism towards Western medications [50] or the stronger beliefs among Asians compared to Europeans that medications are inherently harmful [51]. On the contrary, people's medication concerns in the UK may be more closely linked with their perceived sensitivity (INAS‐Sensitivity to medication). According to the Perceptions and Practicalities Approach, perceived sensitivity to medication is inter‐connected with beliefs about medications [37]. PwT2D who feel that they are more sensitive to medication effects are likely to have negative medication beliefs and low adherence to their medications [37]. This insight on the distinctive factor offers a nuanced understanding of the different types of concerns PwT2D may have towards their medications and also reinforces the more prominent role that medication concern may play in influencing adherence compared to necessity beliefs across countries.

Interestingly, INAS‐Testing treatment, which examined people's tendency to test their level of medication intake was non‐significantly associated with baseline medication adherence (MARS‐5) but was significantly associated with baseline HbA1c in Singapore. One possible explanation was that MARS‐5, which was designed to capture a wide range of nonadherent behaviours [25], uses more general phrasing (e.g., *I stop taking them for a while*). Conversely, INAS, which aims to investigate specific drivers uses more detailed phrasing (e.g., *I sometimes stop taking my diabetes medication to see if I can do without taking my diabetes medication*). This greater specificity in phrasing may help participants better recall their medication‐taking behaviour, resulting in significant relationships found with HbA1c in Singapore. Additionally, participants in Singapore may have a higher tendency to provide socially desirable survey responses than in the UK, as suggested in another study [52]. This may be related to Singapore's more collectivistic culture, which places greater value on others' views than UK's more individualistic culture [49]. This may also explain the absence of a significant association between MARS‐5 and HbA1c in Singapore, as opposed to the significant association found between MARS‐5 and HbA1c in the UK. Understanding the cultural contexts and the design of measurement tools are necessary to capture the subtleties of medication adherence across different countries.

### 4.3 Strengths and Limitations

To our knowledge, this is the first study exploring the common and distinctive factors associated with medication adherence and HbA1c among PwT2D in an Asian and a Western country. Using standardised questionnaires allowed for the pooling of data to identify common factors across countries, which were rarely investigated in current literature. The common factors identified could be prioritised in future personalised interventions, alongside country‐specific distinctive factors that were also highlighted in our study. This approach facilitates the development of personalised interventions that are both broadly applicable and locally relevant.

This study relied on self‐report adherence due to restricted access to prescription refill records. While a self‐report measure may be susceptible to social desirability bias and ceiling effects, mitigating strategies were taken, such as adopting non‐judgemental, neutral introductory statements to encourage openness in responses. The use of validated questionnaires and HbA1c enabled us to examine a wide range of psychological and physiological measures and strengthened the robustness of our study. The insights on these modifiable factors support the development of actionable interventions tailored to these specific factors.

Similarly, understanding potential mediation pathways between medication beliefs and illness perceptions enables intervention designers to target these mechanisms of change, thereby improving the effectiveness of adherence interventions. For example, knowing that INAS‐Resisting illness is associated with medication adherence directly and indirectly through BMQ‐Concern in the Singapore sample, future interventions should target both INAS‐Resisting illness and BMQ‐Concern to improve adherence effectively. Our mediation model using baseline data reinforces the potential role of medication concerns as a mediator, albeit not definitive as the longitudinal mediation analyses lacked sufficient power. Nonetheless, the retrospective power calculation offered assurance that the baseline mediation models had enough statistical power. Future investigation may explore how illness perceptions and medication beliefs interact and affect adherence over time in bigger sample sizes with sufficient statistical power.

Our longitudinal analysis offered greater prospective associations of the predictors than our cross‐sectional analysis. However, there were only two timepoints in our longitudinal study due to resource and time constraints. We could account for the effect of repeated measures within individuals over time but were unable to investigate non‐linear changes in medication adherence. Additionally, loss to follow‐up is a limitation in longitudinal studies. About 44% of the participants were not followed up in this study and this was mainly because they did not attend their follow‐up appointment (11%), they were discharged by the endocrine clinic or diabetes team (8%), and they were hospitalised between baseline and follow‐up (8%). Therefore, longitudinal study with more follow‐up timepoints and bigger sample sizes would be advisable to establish stronger causal inferences where resources permit.

The study's ability to comprehensively assess and account for chronic diseases in the analyses was limited by incomplete and restricted medical records. The lower HbA1c observed in the Singapore sample relative to the UK sample may be due to reasons that are not readily evident. Given that medication adherence (MARS‐5) is not significantly different between samples, the HbA1c may be influenced by other factors, such as differences in diet, participant characteristics and treatment approach at a tertiary setting in Singapore and an intermediate setting in the UK. While some variables showed statistically significant difference between samples, the differences may not be clinically significant, for example, the mean age of 58 in Singapore versus 61 in the UK. Nonetheless, relevant sociodemographic and clinical covariates were controlled in the analyses to facilitate robust investigation of factors across samples.

Other contextual factors, such as different data collection periods, healthcare delivery models and availability of medications across countries may have affected the study results. For example, data collection took place during and shortly after the COVID‐19 pandemic in Singapore, but entirely after the pandemic in the UK. Earlier studies found that 24%–30% of the people with diabetes in Singapore [53] and the UK [54] changed their diabetes medication‐taking behaviour during the pandemic. Given that the earlier studies were both cross‐sectional, it is unclear how differences in the data collection periods during and after the pandemic may have affected the PwT2D in both countries. These contextual factors are complex, dynamic, and potential confounders. Although they could not be fully accounted for in the statistical analyses, country was adjusted for in the combined sample analyses and individual samples were analysed separately to mitigate potential biases. Future research could enhance robustness by incorporating more time‐varying covariates, such as dietary changes, or by stratifying participants based on their profile, such as their comorbidities, which we had limited data on. This approach requires more resources to achieve larger sample sizes for adequate statistical power but can yield deeper insights into the dynamic factors influencing diabetes management.

To translate our findings into practical interventions, identified adherence factors can be mapped to conceptual frameworks, such as the Theoretical Domain Framework, that link these factors to behaviour change techniques that are more likely to address them effectively [55]. This mapping could increase the success of the interventions through its theoretical foundation as well as allowing these to be more personalised.

The study findings from these two multiethnic and multicultural countries may offer some international insights. Common factors could be prioritised first, alongside distinctive factors that are relevant to the target participants. For example, PwT2D across different countries and contexts may benefit from interventions improving perceived personal control over diabetes (BIPQ3), a common factor. For PwT2D in the UK, interventions may target common factors together with perceived sensitivity to medication (INAS), a distinctive factor. Future research could draw on cross‐cultural theories, such as Hofstede's Cultural Dimensions Theory, to investigate how cultural differences may affect individuals' perceptions, psychology, and medication adherence [56].

## 5 Conclusion

Our study identified common and distinctive modifiable factors associated with medication adherence and glycaemic level in Singapore and the UK. Common factors, such as resistance to illness and perceived personal control, may affect medication adherence or glycaemic level. Distinctive factors associated with medication adherence or glycaemic level include perceived sensitivity to medication and testing treatment. Our findings could inform the development of broadly applicable and locally relevant personalised interventions aimed at addressing the factors identified.

## Author Contributions

**Vivien Teo:** conceptualization, data curation, formal analysis, investigation, methodology, project administration, writing – original draft, review and editing. **John Weinman and Kai Zhen Yap:** conceptualization, methodology, supervision, writing – review and editing. **Shaun Eric Lopez, Anna Hodgkinson and Mark Chamley:** project administration, writing – review and editing.

## Funding

This study did not receive any specific grant from funding agencies in the public, commercial or not‐for‐profit sectors.

## Ethics Statement

This study was approved by the Institutional Review Board and Ethics Committees, as detailed in the Methodology.

## Consent

Patient written informed consent was obtained.

## Conflicts of Interest

The authors declare no conflicts of interest.

## Supporting information


**Data S1:** edm270190‐sup‐0001‐DataS1.docx.

## Data Availability

The data supporting the findings of this study are available within the article and its Supporting Information—S1.
