# Supplementary material for: Understanding Medication Adherence and Glycaemic Level in People With Type 2 Diabetes Across Countries: A Cross‐Sectional and Longitudinal Analysis of Medication Beliefs, Illness Perceptions, Resistance to Illness and Mood
Source: Endocrinol Diabetes Metab. 2026 Mar 3;9(2):e70190. doi: 10.1002/edm2.70190 (PMC12956836; doi:10.1002/edm2.70190)
Supplement: Supplementary file 1 — Data S1: edm270190‐sup‐0001‐DataS1.docx. [file EDM2-9-e70190-s001.docx]

# Appendix 1: Measurement invariance of the INAS (UK factor structure)

| Model | RMSEA | ΔRMSEA | SRMR | ΔSRMR | CFI | ΔCFI | TLI | ΔTLI | LR test p-value |
| --- | --- | --- | --- | --- | --- | --- | --- | --- | --- |
| Configural | 0.115 | N/A | 0.047 | N/A | 0.914 | N/A | 0.897 | N/A | N/A |
| Metric | 0.116 | 0.001 | 0.069 | 0.022 | 0.909 | -0.005 | 0.896 | -0.001 | <0.001 |
| Scalar | 0.113 | -0.003 | 0.069 | 0.000 | 0.908 | -0.001 | 0.901 | 0.005 | 0.062 |
| Strict | 0.131 | 0.018 | 0.077 | 0.008 | 0.869 | -0.039 | 0.867 | -0.034 | <0.001 |

CFI: Comparative fit index. LR test: Likelihood ratio test. N/A: Non-applicable. RMSEA: Root mean square error of approximation. SRMR: Standardised Root Mean Square Residual. TLI: Tucker Lewis index.

# Appendix 2: Singapore INAS and guide to using the questionnaire

**Guide to using this questionnaire**

**Suggest using Singapore factor structure in the clinical setting**

Reason: It is more specific to the local context

Sum the scores for the items below to calculate the total score for each INAS factor

| INAS factor | Item |
| --- | --- |
| Resisting illness and medication | 14, 15, 16, 17, 18, 20, 22 |
| Sensitivity to medication | 7, 8, 9, 10, 13 |
| Testing treatment | 1, 2, 5, 6 |
| Inconvenience | 19, 21 |

**Suggest using UK factor structure in the research setting for cross-country comparison**

Reason: It demonstrated measurement invariance for comparative purpose (Appendix 1)

Sum the scores for the items below to calculate the total score for each INAS factor

| INAS factor | Item |
| --- | --- |
| Resisting illness | 15, 16, 17, 18, 19, 20, 21 |
| Resisting medication | 8, 11, 12, 13, 14 |
| Testing treatment | 1, 2, 5 |
| Sensitivity to medication | 7, 9, 10 |

**YOU AND YOUR MEDICINE (Intentional nonadherence scale, INAS)**

People have different experiences when taking diabetes medicines and use diabetes medicines in their own way. We are interested in your view and experience to better support you in taking your diabetes medicines.

All the information you provide is confidential. Your response will not affect your medical care. **There are no right or wrong answers to these questions – an answer is correct if it is true for you.**

Please choose an answer that best fits you in the past 3 months by ticking (√) one box for each statement below.

| **I sometimes stop taking my diabetes medicine in the past 3 months** | | Strongly disagree | Disagree | Neutral | Agree | Strongly agree |
| --- | --- | --- | --- | --- | --- | --- |
| 1 | To see if my diabetes is still there |  |  |  |  |  |
| 2 | To see if I can do without taking my diabetes medicine |  |  |  |  |  |
| 3 | Because I am not convinced that my diabetes medicine is really right for me |  |  |  |  |  |
| 4 | Because I am not sure that my healthcare provider chose the right diabetes medicine for me |  |  |  |  |  |
| 5 | To see if I really need to take my diabetes medicine |  |  |  |  |  |
| 6 | To give my body a break from my diabetes medicine |  |  |  |  |  |
| 7 | Because my diabetes medicine is too strong for my body |  |  |  |  |  |
| 8 | Because I don’t like my diabetes medicine to build up in my body |  |  |  |  |  |
| 9 | Because my body is sensitive to effects of my diabetes medicine |  |  |  |  |  |
| 10 | Because I don’t like the side effects |  |  |  |  |  |
| 11 | Because I don’t like chemicals in my body |  |  |  |  |  |

| **I sometimes stop taking my diabetes medicine in the past 3 months** | | Strongly disagree | Disagree | Neutral | Agree | Strongly agree |
| --- | --- | --- | --- | --- | --- | --- |
| 12 | Because my diabetes medicine may affect my body’s own healing process |  |  |  |  |  |
| 13 | Because I think my diabetes medicine dose is too high for me |  |  |  |  |  |
| 14 | Because I think my diabetes medicine might become less effective over time with regular use. |  |  |  |  |  |
| 15 | Because I worry about becoming dependent on my diabetes medicine |  |  |  |  |  |
| 16 | Because I want to think of myself again as a healthy person without diabetes |  |  |  |  |  |
| 17 | Because it is good not to have to remember to take my diabetes medicine |  |  |  |  |  |
| 18 | Because my diabetes medicine reminds me that I have diabetes |  |  |  |  |  |
| 19 | Because it is inconvenient to take my diabetes medicine all the time |  |  |  |  |  |
| 20 | Because I want to lead a normal life again without having to take diabetes medicine |  |  |  |  |  |
| 21 | Because the timing to take the diabetes medicine doesn’t fit my daily schedule. |  |  |  |  |  |
| 22 | Because I don’t think my diabetes medicine is worth it |  |  |  |  |  |

| What are the main reasons for not taking your diabetes medicine as prescribed? |
| --- |
|  |

# Appendix 3: Multivariate Regression on Factors associated with Baseline MARS-5 and HbA1c in Singapore (n=290)

| **Outcome** | **Baseline MARS-5** | | **Baseline HbA1c** | |
| --- | --- | --- | --- | --- |
| **Variables** | **Coefficient** | **p-value** | **Coefficient** | **p-value** |
| **Age** | **0.037** | **0.001** | NA | NA |
| Years of diabetes | Reference: ≥20 years | | NA | |
| Unspecified record | 0.122 | 0.726 | NA | NA |
| ≤ 1 year | 0.406 | 0.499 | NA | NA |
| >1 year, ≤ 5years | -0.273 | 0.575 | NA | NA |
| > 5 years, < 10 years | 0.835 | 0.055 | NA | NA |
| 10-19 years | 0.437 | 0.192 | NA | NA |
| Ethnicity | NA | | Reference: Chinese | |
| Malay | NA | NA | -1.180 | 0.527 |
| Indian | NA | NA | -0.133 | 0.623 |
| Others | NA | NA | -1.191 | 0.113 |
| Medication type | Reference: Oral only | | Reference: Oral only | |
| Injectables only | 1.256 | 0.105 | 0.795 | 0.210 |
| **Oral + Injectable** | -0.174 | 0.504 | **1.209** | **<0.001** |
| MARS-5 | NA | NA | -0.058 | 0.181 |
| **INAS-Resisting illness** | **-0.110** | **0.011** | 0.034 | 0.268 |
| INAS-Resisting medication | 0.070 | 0.334 | NA | NA |
| **INAS-Testing treatment** | -0.062 | 0.449 | **0.146** | **0.028** |
| INAS-Sensitivity to medication | -0.124 | 0.189 | -0.105 | 0.119 |
| **BMQ-Concern** | **-0.101** | **0.006** | 0.003 | 0.931 |
| BIPQ1-Consequence | NA | NA | -0.011 | 0.818 |
| BIPQ2-Timeline | NA | NA | 0.059 | 0.154 |
| **BIPQ3-Personal control** | NA | NA | **-0.132** | **0.006** |
| BIPQ4-Treatment control | 0.041 | 0.555 | NA | NA |
| **Outcome** | **Baseline MARS-5** | | **Baseline HbA1c** | |
| **Variables** | **Coefficient** | **p-value** | **Variables** | **Coefficient** |
| BIPQ5-Identity | NA | NA | -0.003 | 0.943 |
| BIPQ6-Concerns | NA | NA | 0.029 | 0.499 |
| BIPQ7-Understanding | 0.080 | 0.157 | NA | NA |
| BIPQ8-Emotional response | NA | NA | 0.069 | 0.098 |
| PHQ-2 | NA | NA | 0.016 | 0.789 |

BIPQ: Brief Illness Perception Questionnaire; BMQ: Beliefs about Medicine Questionnaires; INAS: Intentional Nonadherence Scale; MARS-5: Medication adherence report scale-5; NA: Non-applicable; PHQ-2: Patient Health Questionnaire-2.

# Appendix 4: Multivariate Regression on Factors associated with Baseline MARS-5 and HbA1c in the UK (n=260)

| **Outcome** | **Baseline MARS-5** | | **Baseline HbA1c** | |
| --- | --- | --- | --- | --- |
| **Variables** | **Coefficient** | **p-value** | **Coefficient** | **p-value** |
| **Age** | **0.047** | **0.003** | NA | |
| Ethnicity | Reference: White | | NA | NA |
| Black/ Black British | -0.046 | 0.920 | NA | NA |
| Asian/ Asian British | 0.705 | 0.324 | NA | NA |
| Mixed | 0.288 | 0.722 | NA | NA |
| Others | 0.689 | 0.284 | NA | NA |
| Declined to answer | -0.149 | 0.941 | NA | NA |
| Relationship status | Reference: Single | | NA | |
| Married | -0.299 | 0.467 | NA | NA |
| Separated/ divorced/ widowed | -0.694 | 0.181 | NA | NA |
| Others | -0.485 | 0.629 | NA | NA |
| Declined to answer | -3.288 | 0.386 | NA | NA |
| **Highest education level** | Reference: No formal education | | Reference: No formal education | |
| **Primary school/ lower** | 0.431 | 0.781 | **-23.145** | **0.034** |
| **Secondary school** | 0.172 | 0.905 | **-19.863** | **0.048** |
| **A-level/diploma** | 0.302 | 0.837 | **-21.860** | **0.031** |
| **Degree/higher** | -0.687 | 0.635 | **-25.286** | **0.012** |
| **Declined to answer*** | 0 (omitted) | - | **-58.077** | **0.009** |
| Medication type | Reference: Oral only | | Reference: Oral only | |
| Injectables only | -0.545 | 0.548 | 4.678 | 0.464 |
| **Oral + Injectable** | **-0.921** | **0.023** | 2.172 | 0.434 |
| **MARS-5** | NA | NA | **-1.020** | **0.040** |
| INAS-Resisting illness | 0.032 | 0.674 | 0.186 | 0.604 |
| INAS-Resisting medication | 0.032 | 0.792 | NA | NA |
| **Outcome** | **Baseline MARS-5** | | **Baseline HbA1c** | |
| **Variables** | **Coefficient** | **p-value** | **Variables** | **Coefficient** |
| INAS-Testing treatment | -0.159 | 0.271 | NA | NA |
| **INAS-Sensitivity to medication** | **-0.262** | **0.019** | 0.678 | 0.335 |
| BMQ-Concern | -0.042 | 0.395 | NA | NA |
| BIPQ1-Consequence | -0.010 | 0.904 | NA | NA |
| BIPQ2-Timeline | 0.106 | 0.192 | NA | NA |
| **BIPQ3-Personal control** | 0.102 | 0.283 | **-1.803** | **0.001** |
| BIPQ4-Treatment control | 0.018 | 0.868 | NA | NA |
| BIPQ7-Understanding | 0.115 | 0.272 | NA | NA |
| BIPQ8-Emotional response | -0.017 | 0.779 | 0.634 | 0.090 |

BIPQ: Brief Illness Perception Questionnaire; BMQ: Beliefs about Medicine Questionnaires; INAS: Intentional Nonadherence Scale; MARS-5: Medication adherence report scale-5; NA: Non-applicable; PHQ-2: Patient Health Questionnaire-2. *Response category was omitted due to collinearity.

# Appendix 5: Univariate Regression on Factors associated with Baseline MARS-5 in the Combined, Singapore and UK sample

| Variables | Combined sample (n=550) | | Singapore (n=290) | | UK (n=260) | |
| --- | --- | --- | --- | --- | --- | --- |
|  | Coefficient | p-value | Coefficient | p-value | Coefficient | p-value |
| Age | **0.042** | **<0.001** | **0.038** | **0.005** | **0.042** | **0.008** |
| Sex | Reference: Male | | Reference: Male | | Reference: Male | |
| Female | 0.000 | 1.000 | 0.000 | 1.000 | 0.000 | 1.000 |
| Ethnicity | Reference: White | | Reference: Chinese | | Reference: White | |
| Chinese | 0.000 | 1.000 | - | - | - | - |
| Malay | 0.000 | 1.000 | 1.000 | 0.338 |  |  |
| Indian | -1.000 | 0.059 | 1.000 | 0.359 | - | - |
| Others | 0.000 | 1.000 | 0.000 | 1.000 | 0.000 | 1.000 |
| Black/ Black British | **-1.000** | **0.020** | - | - | **-1.000** | **0.023** |
| Asian/ Asian British | 0.000 | 1.000 | - | - | 0.000 | 1.000 |
| Mixed | 0.000 | 1.000 | - | - | 0.000 | 1.000 |
| Declined to answer | -2.000 | 0.241 | - | - | -2.000 | 0.252 |
| Highest education level | Reference: No formal education | | Reference: No formal education | | Reference: No formal education | |
| Primary school/ lower | -1.000 | 0.569 | -1.000 | 0.747 | -1.000 | 0.595 |
| Secondary school | -1.000 | 0.560 | -1.000 | 0.745 | -1.000 | 0.607 |
| A-level/diploma | -1.000 | 0.564 | -2.000 | 0.518 | 0.000 | 1.000 |
| Degree/higher | -2.000 | 0.249 | -1.000 | 0.748 | -2.000 | 0.293 |
| Declined to answer | -8.000 | 0.055 | - | - | **-8.000** | **<0.001** |
| Relationship status | Reference: Single | | Reference: Single | | Reference: Single | |
| Married | 0.000 | 1.000 | 0.000 | 1.000 | 0.000 | 1.000 |
| Separated/ divorced/ widowed | 0.000 | 1.000 | 0.000 | 1.000 | 0.000 | 1.000 |
| Others | 0.000 | 1.000 | 1.000 | 0.648 | 0.000 | 1.000 |
| Declined to answer | -7.000 | 0.095 | - | - | **-7.000** | **0.031** |
| Variables | Combined sample (n=550) | | Singapore (n=290) | | UK (n=260) | |
|  | Coefficient | p-value | Coefficient | p-value | Coefficient | p-value |
| Manage own medication | Reference: No | | Reference: No | | Reference: No | |
| Yes | 0.000 | 1.000 | -1.000 | 0.257 | 0.000 | 1.000 |
| Years of diabetes | Reference: ≥20 years | | Reference: ≥20 years | | Reference: ≥20 years | |
| Unspecified record | **-1.000** | **0.040** | **-1.000** | **0.017** | -1.000 | 0.378 |
| ≤ 1 year | -1.000 | 0.170 | -1.000 | 0.148 | 0.000 | 1.000 |
| >1 year, ≤ 5years | -1.000 | 0.085 | -1.000 | 0.079 | 0.000 | 1.000 |
| > 5 years, < 10 years | 0.000 | 1.000 | 0.000 | 1.000 | 0.000 | 1.000 |
| 10-19 years | 0.000 | 1.000 | 0.000 | 1.000 | 0.000 | 1.000 |
| Medication type | Reference: Oral only | | Reference: Oral only | | Reference: Oral only | |
| Injectables only | 0.000 | 1.000 | 1.000 | 0.378 | 0.000 | 1.000 |
| Oral + Injectable | 0.000 | 1.000 | 0.000 | 1.000 | 0.000 | 1.000 |
| INAS-Resisting illness | **-0.167** | **<0.001** | **-0.176** | **<0.001** | **-0.167** | **<0.001** |
| INAS-Resisting medication | **-0.235** | **<0.001** | **-2.000** | **<0.001** | **-0.250** | **<0.001** |
| INAS-Testing treatment | **-0.333** | **<0.001** | **-0.333** | **<0.001** | **-0.571** | **<0.001** |
| INAS-Sensitivity to medication | **-0.333** | **<0.001** | **-0.333** | **<0.001** | **-0.333** | **<0.001** |
| BMQ-Necessity | 0.000 | 1.000 | 0.000 | 1.000 | 0.000 | 1.000 |
| BMQ-Concern | **-0.143** | **<0.001** | **-0.154** | **0.001** | **-0.133** | **0.014** |
| BIPQ1-Consequence | 0.000 | 1.000 | 0.000 | 1.000 | **-0.200** | **0.010** |
| BIPQ2-Timeline | -1.000 | 0.102 | 0.000 | 1.000 | **0.167** | **0.027** |
| BIPQ3-Personal control | **0.167** | **0.003** | 0.125 | 0.121 | **0.200** | **0.025** |
| BIPQ4-Treatment control | **0.333** | **<0.001** | **0.333** | **<0.001** | **0.333** | **0.001** |
| BIPQ5-Identity | 0.000 | 1.000 | 0.000 | 1.000 | 0.000 | 1.000 |
| BIPQ6-Concerns | 0.000 | 1.000 | 0.000 | 1.000 | 0.000 | 1.000 |
| BIPQ7- Understanding | **0.143** | **0.033** | **0.167** | **0.018** | **0.200** | **0.023** |
| BIPQ8-Emotional response | **-0.143** | **<0.001** | 0.000 | 1.000 | **-0.200** | **<0.001** |
| Variables | Combined sample (n=550) | | Singapore (n=290) | | UK (n=260) | |
|  | Coefficient | p-value | Coefficient | p-value | Coefficient | p-value |
| PHQ-2 | -0.167 | 0.066 | -0.167 | 0.190 | -0.167 | 0.253 |

BIPQ: Brief Illness Perception Questionnaire; BMQ: Beliefs about Medicine Questionnaires; INAS: Intentional Nonadherence Scale; MARS-5: Medication adherence report scale-5; NA: Non-applicable; PHQ-2: Patient Health Questionnaire-2.

# Appendix 6: Univariate Regression on Factors associated with Baseline HbA1c in the Combined, Singapore and UK sample

| Variables | Combined sample (n=550) | | Singapore (n=290) | | UK (n=260) | |
| --- | --- | --- | --- | --- | --- | --- |
|  | Coefficient | p-value | Coefficient | p-value | Coefficient | p-value |
| Age | 0.000 | 0.952 | -0.011 | 0.219 | 0.015 | 0.894 |
| Sex | Reference: Male | | Reference: Male | | Reference: Male | |
| Female | 0.217 | 0.174 | 0.130 | 0.534 | 0.377 | 0.882 |
| Ethnicity | Reference: White | | Reference: Chinese | | Reference: White | |
| Chinese | **-1.002** | **<0.001** | - | - | - | - |
| Malay | -0.523 | 0.130 | 0.479 | 0.099 | - | - |
| Indian | **-0.841** | **0.010** | 0.162 | 0.549 | - | - |
| Others | 0.205 | 0.583 | **1.528** | **0.048** | 1.595 | 0.727 |
| Black/ Black British | 0.216 | 0.414 | - | - | 2.400 | 0.432 |
| Asian/ Asian British | -0.295 | 0.490 | - | - | -3.166 | 0.519 |
| Mixed | 0.120 | 0.818 | - | - | 1.452 | 0.809 |
| Declined to answer | 0.205 | 0.845 | - | - | 2.095 | 0.863 |
| Highest education | Reference: No formal education | | Reference: No formal education | | Reference: No formal education | |
| Primary school/ lower | **-2.148** | **0.012** | -1.720 | 0.319 | -20.583 | 0.068 |
| Secondary school | **-2.174** | **0.009** | -2.129 | 0.215 | -17.137 | 0.100 |
| A-level/diploma | **-2.330** | **0.006** | -2.231 | 0.196 | **-21.275** | **0.044** |
| Degree/higher | **-2.151** | **0.011** | -2.297 | 0.186 | **-22.174** | **0.034** |
| Declined to answer | -2.960 | 0.144 | - | - | -33.750 | 0.139 |
| Relationship status | Reference: Single | | Reference: Single | | Reference: Single | |
| Married | -0.275 | 0.134 | 0.108 | 0.667 | -2.427 | 0.403 |
| Separated/ divorced/ widowed | 0.314 | 0.220 | 0.379 | 0.317 | 2.743 | 0.454 |
| Others | 0.393 | 0.495 | -0.858 | 0.487 | 4.000 | 0.576 |
| Declined to answer | -0.879 | 0.635 | - | - | -14.444 | 0.483 |
| Variables | Combined sample (n=550) | | Singapore (n=290) | | UK (n=260) | |
|  | Coefficient | p-value | Coefficient | p-value | Coefficient | p-value |
| Manage own medication | Reference: No |  | Reference: No | | Reference: No | |
| Yes | -0.068 | 0.868 | 0.010 | 0.983 | -10.077 | 0.233 |
| Years of diabetes | Reference: ≥20 years | | Reference: ≥20 years | | Reference: ≥20 years | |
| Unspecified record | **-0.574** | **0.027** | -0.251 | 0.391 | 0.167 | 0.980 |
| ≤ 1 year | -0.644 | 0.097 | -0.722 | 0.138 | -4.167 | 0.517 |
| >1 year, ≤ 5years | -0.451 | 0.145 | -0.763 | 0.057 | -0.225 | 0.964 |
| > 5 years, < 10 years | -0.145 | 0.593 | -0.318 | 0.371 | 1.020 | 0.812 |
| 10-19 years | 0.042 | 0.840 | 0.227 | 0.423 | -1.86 | 0.553 |
| Medication type | Reference: Oral only | | Reference: Oral only | | Reference: Oral only | |
| Injectables only | **1.119** | **0.014** | **1.247** | **0.052** | 6.481 | 0.328 |
| Oral + Injectable | **0.757** | **<0.001** | **1.181** | **<0.001** | 2.168 | 0.447 |
| MARS-5 | **-0.128** | **<0.001** | **-0.110** | **0.009** | **-1.474** | **0.001** |
| INAS-Resisting illness | **0.043** | **0.009** | **0.061** | **0.002** | **0.580** | **0.039** |
| INAS-Resisting medication | 0.024 | 0.303 | 0.042 | 0.151 | 0.315 | 0.413 |
| INAS-Testing treatment | **0.072** | **0.050** | **0.113** | **0.011** | 0.711 | 0.268 |
| INAS-Sensitivity to medication | **0.103** | **0.003** | **0.087** | **0.054** | **1.393** | **0.012** |
| BMQ-Necessity | 0.038 | 0.076 | 0.015 | 0.582 | 0.001 | 0.999 |
| BMQ-Concern | **0.046** | **0.028** | **0.070** | **0.015** | 0.328 | 0.297 |
| BIPQ1-Consequence | **0.072** | **0.014** | **0.109** | **0.004** | -0.341 | 0.475 |
| BIPQ2-Timeline | 0.037 | 0.243 | **0.077** | **0.052** | -0.334 | 0.529 |
| BIPQ3-Personal control | **-0.132** | **<0.001** | **-0.131** | **0.009** | **-2.037** | **<0.001** |
| BIPQ4-Treatment control | -0.010 | 0.812 | -0.042 | -0.790 | -0.751 | 0.246 |
| BIPQ5-Identity | **0.095** | **0.001** | **0.078** | **0.042** | 0.326 | 0.492 |
| BIPQ6-Concerns | **0.099** | **0.001** | **0.097** | **0.010** | 0.548 | 0.276 |
| BIPQ7- Understanding | -0.006 | 0.883 | -0.023 | 0.620 | -0.284 | 0.656 |
| BIPQ8-Emotional response | **0.098** | **<0.001** | **0.092** | **0.007** | **0.982** | **0.009** |
| Variables | Combined sample (n=550) | | Singapore (n=290) | | UK (n=260) | |
|  | Coefficient | p-value | Coefficient | p-value | Coefficient | p-value |
| PHQ-2 | 0.084 | 0.058 | **0.141** | **0.008** | 0.637 | 0.398 |

BIPQ: Brief Illness Perception Questionnaire; BMQ: Beliefs about Medicine Questionnaires; INAS: Intentional Nonadherence Scale; MARS-5: Medication adherence report scale-5; NA: Non-applicable; PHQ-2: Patient Health Questionnaire-2.

# Appendix 7: Sensitivity analysis comparing participants who were and were not followed up in 3-6 months in the combined sample

| Variable | Number (%) or Mean ± Standard deviation or Median (Interquartile range) | | |
| --- | --- | --- | --- |
|  | No follow-up (n=241) | Follow-up (n= 309) | p-value |
| Age | 59.7 ± 12.2 | 59.1 ± 11.5 | 0.560 |
| Sex |  |  | 0.402 |
| Male | 131 (54.4) | 179 (57.9) |  |
| Female | 110 (45.6) | 130 (42.1) |  |
| Ethnicity |  |  | **0.005** |
| White | 36 (14.9) | 38 (12.3) |  |
| Black/ Black British | 66 (27.4) | 52 (16.8) |  |
| Asian/ Asian British | 9 (3.7) | 14 (4.5) |  |
| Mixed | 9 (3.7) | 5 (1.6) |  |
| Others | 16 (6.6) | 17 (5.5) |  |
| Chinese - Singapore | 69 (28.6) | 124 (40.1) |  |
| Malay – Singapore | 16 (6.6) | 26 (8.4) |  |
| Indian - Singapore | 17 (7.1) | 33 (10.7) |  |
| Declined to answer | 3 (1.2) | 0 (0) |  |
| Highest education |  |  | 0.799 |
| No formal education | 3 (1.2) | 2 (0.6) |  |
| Primary school/ lower | 32 (13.3) | 42 (13.6) |  |
| O-level/ Secondary school | 103 (42.7) | 125 (40.5) |  |
| A-level/diploma | 52 (21.6) | 74 (23.9) |  |
| Degree/higher | 50 (20.8) | 66 (21.4) |  |
| Declined to answer | 1 (0.4) | 0 (0) |  |
| Variable | Number (%) or Mean ± Standard deviation or Median (Interquartile range) | | |
|  | No follow-up (n=241) | Follow-up (n= 309) | p-value |
| Relationship status |  |  | **0.021** |
| Single | 80 (33.2) | 71 (23.0) |  |
| Married | 119 (49.4) | 189 (61.2) |  |
| Separated/ divorced/ widowed | 34 (14.1) | 45 (14.6) |  |
| Others | 7 (2.9) | 4 (1.3) |  |
| Declined to answer | 1 (0.4) | 0 (0) |  |
| Manage own medication |  |  | 0.928 |
| Yes | 232 (96.3) | 297 (96.1) |  |
| No | 9 (3.7) | 12 (3.9) |  |
| Years of diabetes |  |  | 0.779 |
| No records | 35 (14.5) | 46 (14.9) |  |
| ≤ 1 year | 9 (3.7) | 18 (5.8) |  |
| >1 year, ≤ 5 years | 24 (10.0) | 24 (7.8) |  |
| >5 years, <10 years | 33 (13.7) | 36 (11.7) |  |
| 10-19 years | 81 (33.6) | 107 (34.6) |  |
| ≥ 20 years | 59 (24.5) | 78 (25.2) |  |
| Medication type |  |  | 0.707 |
| Oral only | 74 (30.7) | 88 (28.5) |  |
| Injectables only | 9 (3.7) | 9 (2.9) |  |
| Oral + injectables | 158 (65.6) | 212 (68.6) |  |
| Number of chronic diseases* | 3.0 ± 1.5 | 3.0 ± 1.4 | 0.652 |
| Baseline HbA1c (mmol/mol) | 77.4 ± 23.1 | 78.6 ± 17.1 | 0.656 |
| Baseline HbA1c (%) | 8.9 ± 2.1 | 8.7 ± 1.7 | 0.181 |
| Variable | Number (%) or Mean ± Standard deviation or Median (Interquartile range) | | |
|  | No follow-up (n=241) | Follow-up (n= 309) | p-value |
| VAS | 100 (100-80) | 100 (100-80) | 0.919 |
| INAS-Resisting illness | 13.9 ± 4.8 | 13.9 ± 4.9 | 0.980 |
| INAS-Resisting medication | 9.7 ± 3.5 | 9.7 ± 3.3 | 0.993 |
| INAS-Testing treatment | 5.7 ± 2.3 | 5.7 ± 2.1 | 0.773 |
| INAS-Sensitivity to medication | 6.0 ± 2.4 | 5.8 ± 2.1 | 0.414 |
| Baseline MARS-5 | 24 (25-22) | 24 (25-22) | 0.572 |
| BMQ-Necessity | 16.8 ± 3.9 | 16.6 ± 3.6 | 0.604 |
| BMQ-Concern | 13.4 ± 3.8 | 13.8 ± 3.7 | 0.219 |
| BIPQ1-Consequence | 5.5 ± 2.8 | 5.7 ± 2.6 | 0.357 |
| BIPQ2-Timeline | 7.7 ± 2.5 | 7.8 ± 2.5 | 0.826 |
| BIPQ3-Personal control | 7.0 ± 2.2 | 6.7 ± 2.2 | 0.096 |
| BIPQ4-Treatment control | 7.8 ± 2.0 | 7.9 ± 1.9 | 0.841 |
| BIPQ5-Identity | 5.5 ± 2.9 | 5.3 ± 2.6 | 0.346 |
| BIPQ6-Concerns | 7.4 ± 2.9 | 7.4 ± 2.4 | 0.831 |
| BIPQ7-Understanding | 7.9 ± 2.0 | 7.6 ± 2.1 | 0.065 |
| BIPQ8-Emotional response | 5.2 ± 3.2 | 5.4 ± 3.1 | 0.421 |
| PHQ-2 | 0 (2-0) | 0 (2-0) | 0.916 |

BIPQ: Brief Illness Perception Questionnaire; BMQ: Beliefs about Medicine Questionnaires; INAS: Intentional Nonadherence Scale; MARS-5: Medication adherence report scale-5; PHQ-2: Patient Health Questionnaire-2; VAS: Visual Analogue Scale; *Limited information on chronic diseases

# Appendix 8: Conceptual moderated mediation model with baseline MARS-5 as the outcome


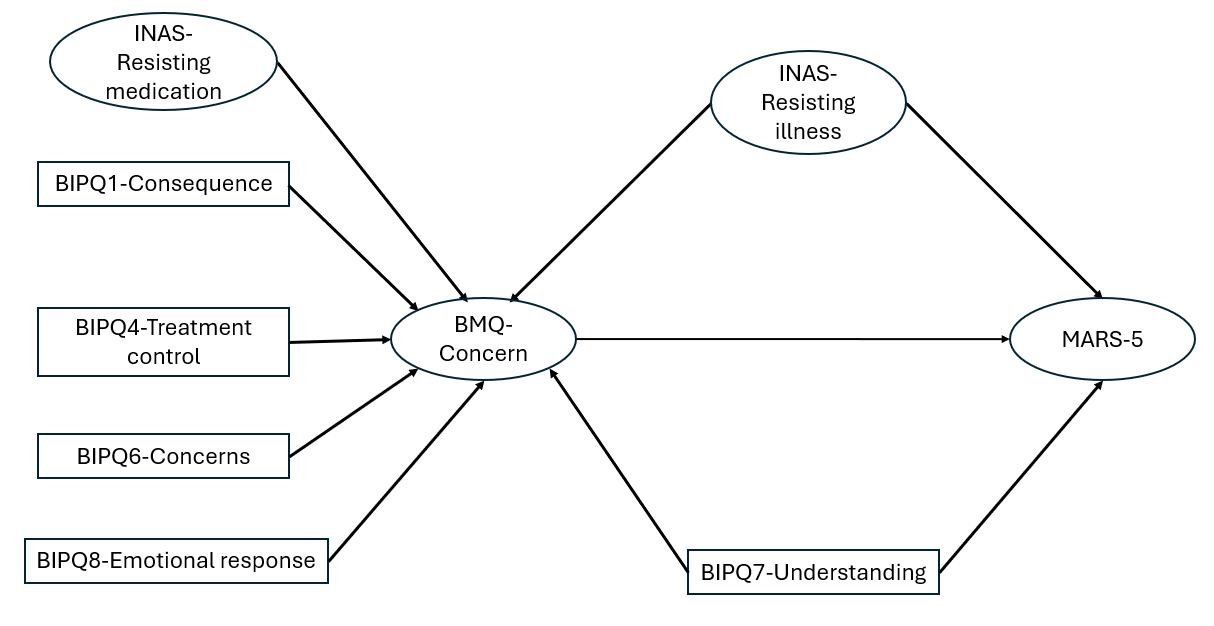


BIPQ: Brief Illness Perception Questionnaire; BMQ: Beliefs about Medicine Questionnaires; INAS: Intentional Nonadherence Scale; MARS-5: Medication adherence report scale-5. Latent variables are shown in circles, while observed variables are shown in rectangles.

# Appendix 9: Univariate and multivariate regressions on Factors associated with BMQ-concern in the combined sample

## Multivariate regression

| Variables | Coefficient | p-value |
| --- | --- | --- |
| Sex | Reference: Male | |
| Female | 0.534 | 0.052 |
| Ethnicity | Reference: White | |
| Black/ Black British | 0.032 | 0.947 |
| Asian/ Asian British | 1.848 | 0.015 |
| Mixed | 0.062 | 0.946 |
| Others | 0.067 | 0.921 |
| Chinese - Singapore | 0.299 | 0.509 |
| Malay - Singapore | 0.372 | 0.556 |
| Indian - Singapore | 1.282 | 0.039 |
| Declined to answer | 2.752 | 0.141 |
| Years of diabetes | Reference: ≥20 years | |
| Unspecified record | 0.307 | 0.513 |
| ≤ 1 year | 0.382 | 0.573 |
| >1 year, ≤ 5years | 0.255 | 0.634 |
| > 5 years, < 10 years | 1.379 | 0.004 |
| 10-19 years | 0.988 | 0.006 |
| Medication type | Reference: Oral only | |
| Injectables only | -0.320 | 0.687 |
| Oral + Injectable | 0.048 | 0.878 |
| **INAS-Resisting illness** | **0.166** | **0.001** |
| INAS-Resisting medication | 0.147 | 0.086 |
| INAS-Testing treatment | -0.083 | 0.400 |
| Variables | Coefficient | p-value |
| INAS-Sensitivity to medication | -0.121 | 0.214 |
| **BIPQ1-Consequence** | **0.295** | **<0.001** |
| **BIPQ4-Treatment control** | **-0.311** | **<0.001** |
| BIPQ5-Identity | 0.012 | 0.843 |
| **BIPQ6-Concerns** | **0.200** | **0.001** |
| **BIPQ7-Understanding** | **-0.166** | **0.020** |
| **BIPQ8-Emotional response** | **0.201** | **<0.001** |
| PHQ-2 | -0.035 | 0.687 |

BIPQ: Brief Illness Perception Questionnaire; BMQ: Beliefs about Medicine Questionnaires; INAS: Intentional Nonadherence Scale; MARS-5: Medication adherence report scale-5; PHQ-2: Patient Health Questionnaire-2

## Univariate regression

| Variables | Coefficient | p-value |
| --- | --- | --- |
| Age | -0.021 | 0.115 |
| Sex | Reference: Male | |
| **Female** | **0.731** | **0.023** |
| Ethnicity | Reference: White | |
| **Black/ Black British** | **1.636** | **0.003** |
| Asian/ Asian British | 3.276 | **<0.001** |
| Mixed | 0.797 | 0.454 |
| **Others** | **2.161** | **0.005** |
| Chinese - Singapore | 0.942 | 0.060 |
| Malay - Singapore | **1.821** | **0.010** |
| Indian - Singapore | **2.997** | **<0.001** |
| Declined to answer | **5.464** | **0.011** |
| Highest education | Reference: No formal education | |
| Primary school/ lower | 0.386 | 0.823 |
| Secondary school | 1.093 | 0.518 |
| A-level/diploma | 1.400 | 0.412 |
| Degree/higher | 0.822 | 0.630 |
| Declined to answer | 7.400 | 0.071 |
| Relationship status | Reference: Single | |
| Married | 0.367 | 0.322 |
| Separated/ divorced/ widowed | -0.306 | 0.556 |
| Others | -1.561 | 0.181 |
| Declined to answer | 6.530 | 0.082 |
| Years of diabetes | Reference: ≥20 years | |
| Unspecified record | 0.545 | 0.298 |
| ≤ 1 year | 0.718 | 0.362 |
| Variables | Coefficient | p-value |
| >1 year, ≤ 5years | 0.364 | 0.562 |
| **> 5 years, < 10 years** | **1.345** | **0.015** |
| **10-19 years** | **1.019** | **0.015** |
| Medication type | Reference: Oral only | |
| Injectables only | -0.259 | 0.781 |
| Oral + Injectable | 0.043 | 0.904 |
| Manage own medication | Reference: No | |
| Yes | 0.043 | 0.959 |
| Number of chronic disease (CD) | -0.057 | 0.614 |
| **INAS-Resisting illness** | **0.261** | **<0.001** |
| **INAS-Resisting medication** | **0.330** | **<0.001** |
| **INAS-Testing treatment** | **0.373** | **<0.001** |
| **INAS-Sensitivity to medication** | **0.366** | **<0.001** |
| **BIPQ1-Consequence** | **0.495** | **<0.001** |
| BIPQ2-Timeline | 0.004 | 0.954 |
| BIPQ3-Personal control | -0.125 | 0.090 |
| **BIPQ4-Treatment control** | **-0.397** | **<0.001** |
| **BIPQ5-Identity** | **0.253** | **<0.001** |
| **BIPQ6-Concerns** | **0.399** | **<0.001** |
| **BIPQ7-Understanding** | **-0.161** | **0.035** |
| **BIPQ8-Emotional response** | **0.432** | **<0.001** |
| **PHQ-2** | **0.380** | **<0.001** |

BIPQ: Brief Illness Perception Questionnaire; BMQ: Beliefs about Medicine Questionnaires; INAS: Intentional Nonadherence Scale; MARS-5: Medication adherence report scale-5; PHQ-2: Patient Health Questionnaire-2

# Appendix 10: Univariate and multivariate regressions on Factors associated with BMQ-concern in Singapore

## Multivariate regression

| Variables | Coefficient | p-value |
| --- | --- | --- |
| Language | Reference: English | |
| Mandarin | -0.610 | 0.180 |
| Ethnicity | Reference: Chinese | |
| Malay | -0.447 | 0.416 |
| Indian | 0.448 | 0.400 |
| Others | -1.462 | 0.288 |
| Medication type | Reference: Oral only | |
| Injectables only | 0.288 | 0.801 |
| Oral + Injectable | -0.402 | 0.294 |
| **INAS-Resisting illness** | **0.147** | **0.019** |
| **INAS-Resisting medication** | **0.279** | **0.009** |
| INAS-Testing treatment | -0.189 | 0.110 |
| INAS-Sensitivity to medication | -0.178 | 0.209 |
| **BIPQ1-Consequence** | **0.222** | **0.006** |
| **BIPQ4-Treatment control** | **-0.310** | **0.002** |
| BIPQ5-Identity | 0.001 | 0.987 |
| BIPQ6-Concerns | 0.157 | 0.057 |
| **BIPQ8-Emotional response** | **0.229** | **0.002** |
| PHQ-2 | 0.166 | 0.121 |

BIPQ: Brief Illness Perception Questionnaire; BMQ: Beliefs about Medicine Questionnaires; INAS: Intentional Nonadherence Scale; MARS-5: Medication adherence report scale-5; PHQ-2: Patient Health Questionnaire-2

## Univariate regression

| Variables | Coefficient | | p-value |
| --- | --- | --- | --- |
| Language | Reference: English | | |
| Mandarin | -1.453 | | 0.001 |
| Age | -0.016 | | 0.357 |
| Sex | Reference: Male | | |
| Female | 0.646 | | 0.126 |
| Ethnicity | Reference: Chinese | | |
| Malay | 0.879 | | 0.129 |
| **Indian** | **2.055** | | **<0.001** |
| Others | 2.455 | | 0.111 |
| Highest education | Reference: No formal education | | |
| Primary school/ lower | -5.429 | | 0.118 |
| Secondary school | -4.326 | | 0.210 |
| A-level/diploma | -3.522 | | 0.309 |
| Degree/higher | -4.258 | | 0.223 |
| Relationship status | Reference: Single | | |
| Married | 0.365 | | 0.476 |
| Separated/ divorced/ widowed | -0.136 | | 0.860 |
| Others | 1.574 | | 0.530 |
| Years of diabetes | Reference: ≥20 years | | |
| Unspecified record | 4.875 | | 0.164 |
| ≤ 1 year | 0.717 | | 0.419 |
| >1 year, ≤ 5years | 0.875 | | 0.154 |
| > 5 years, < 10 years | 0.246 | | 0.727 |
| 10-19 years | 0.825 | | 0.114 |
| Variables | Coefficient | p-value | |
| Medication type | Reference: Oral only | | |
| Injectables only | 0.995 | | 0.467 |
| Oral + Injectable | -0.111 | | 0.804 |
| Manage own medication | Reference: No | | |
| Yes | 0.073 | | 0.937 |
| Number of chronic disease (CD) | -0.032 | | 0.828 |
| **INAS-Resisting illness** | **0.248** | | **<0.001** |
| **INAS-Resisting medication** | **0.328** | | **<0.001** |
| **INAS-Testing treatment** | **0.261** | | **0.004** |
| **INAS-Sensitivity to medication** | **0.365** | | **<0.001** |
| **BIPQ1-Consequence** | **0.465** | | **<0.001** |
| BIPQ2-Timeline | 0.090 | | 0.261 |
| BIPQ3-Personal control | -0.132 | | 0.194 |
| **BIPQ4-Treatment control** | **-0.373** | | **0.001** |
| **BIPQ5-Identity** | **0.247** | | **0.001** |
| **BIPQ6-Concerns** | **0.375** | | **<0.001** |
| BIPQ7-Understanding | -0.099 | | 0.294 |
| **BIPQ8-Emotional response** | **0.480** | | **<0.001** |
| **PHQ-2** | **0.549** | | **<0.001** |

BIPQ: Brief Illness Perception Questionnaire; BMQ: Beliefs about Medicine Questionnaires; INAS: Intentional Nonadherence Scale; MARS-5: Medication adherence report scale-5; PHQ-2: Patient Health Questionnaire-2
